# Supplementary material for: From sequence to enzyme mechanism using multi-label machine learning
Source: BMC Bioinformatics. 2014 May 19;15:150. doi: 10.1186/1471-2105-15-150 (PMC4229970; doi:10.1186/1471-2105-15-150)
Supplement: Additional file 2 — Java code of ml2db. Additional file ml2db_code.tar.gz contains the Java source code to run the multi-label machine learning experiments and save the results to database. The code’s Javadoc is included. [file 1471-2105-15-150-S2.zip › additional file 2/ml2db/utils/doc/uk/ac/standrews/utils/main/stats/PseudoTruncatedPareto.html]

PseudoTruncatedPareto


JavaScript is disabled on your browser.


- Overview
- Package
- Class
- Use
- Tree
- Deprecated
- Index
- Help

- Prev Class
- Next Class

- Frames
- No Frames

- All Classes

- Summary:
- Nested |
- Field |
- Constr |
- Method

- Detail:
- Field |
- Constr |
- Method


uk.ac.standrews.utils.main.stats

## Class PseudoTruncatedPareto

- java.lang.Object
- - cern.colt.PersistentObject
  - - cern.jet.random.AbstractDistribution
    - - cern.jet.random.AbstractContinousDistribution
      - - cern.jet.random.Pareto
        - - uk.ac.standrews.utils.main.stats.TruncatedPareto
          - - uk.ac.standrews.utils.main.stats.PseudoTruncatedPareto

- All Implemented Interfaces:
  :   DoubleFunction, IntFunction, java.io.Serializable, java.lang.Cloneable

  ---

    

  ```
  public class PseudoTruncatedPareto
  extends TruncatedPareto
  ```

  A truncated pareto distribution which stores a big lists of power-law
  extracted random numbers. Used to work around the performance problems of the
  Pareto distribution.

  Version:
  :   4 Aug 2008

  Author:
  :   Luna De Ferrari luna.deferrari-at-ed.ac.uk

  See Also:
  :   Serialized Form

- - ### Constructor Summary

    Constructors

    Constructor and Description

    `PseudoTruncatedPareto(double scale,
    double shape,
    int offset,
    int truncationThreshold,
    int lengthOfPseudoRandomList)`
  - ### Method Summary

    Methods

    | Modifier and Type | Method and Description |
    | --- | --- |
    | `java.lang.Integer[]` | `getPseudoRandomNumbers()` |
    | `int` | `nextPseudoInt()` |

    - ### Methods inherited from class uk.ac.standrews.utils.main.stats.TruncatedPareto

      `getOffset, getPareto, getParetoFrequencies, getTruncationThreshold, nextInt`
    - ### Methods inherited from class cern.jet.random.Pareto

      `cdf, nextDouble, nextDouble, pdf, setState, staticNextDouble, toString`
    - ### Methods inherited from class cern.jet.random.AbstractDistribution

      `apply, apply, clone, makeDefaultGenerator`
    - ### Methods inherited from class java.lang.Object

      `equals, getClass, hashCode, notify, notifyAll, wait, wait, wait`

- - ### Constructor Detail


    - #### PseudoTruncatedPareto

      ```
      public PseudoTruncatedPareto(double scale,
                           double shape,
                           int offset,
                           int truncationThreshold,
                           int lengthOfPseudoRandomList)
      ```

      Parameters:
      :   `scale` - the power law distribution scale
      :   `shape` - the power law distribution shape
      :   `offset` - an offset (all extracted numbers will start from the offset)
      :   `truncationThreshold` - a truncation threshold (no extracted number will be above the
          threshold)
      :   `lengthOfPseudoRandomList` - the length of the list of power-law distributed random numbers
          to be stored
  - ### Method Detail


    - #### getPseudoRandomNumbers

      ```
      public java.lang.Integer[] getPseudoRandomNumbers()
      ```


    - #### nextPseudoInt

      ```
      public int nextPseudoInt()
      ```


- Overview
- Package
- Class
- Use
- Tree
- Deprecated
- Index
- Help

- Prev Class
- Next Class

- Frames
- No Frames

- All Classes

- Summary:
- Nested |
- Field |
- Constr |
- Method

- Detail:
- Field |
- Constr |
- Method
